# Five essential lessons in antimicrobial stewardship and leadership I have learned from running

**DOI:** 10.1017/ash.2025.188

**Published:** 2025-05-19

**Authors:** Miranda So

**Affiliations:** 1 Antimicrobial Stewardship Program, University Health Network, Toronto, Canada; 2 Leslie Dan Faculty of Pharmacy, University of Toronto, Toronto, Canada; 3 Toronto General Hospital Research Institute, Toronto, Canada; 4 Division of Infectious Diseases, Faculty of Medicine, Dentistry and Health Sciences, University of Melbourne, Melbourne, Australia

## Abstract

As an antimicrobial stewardship pharmacist and an avid runner, I identified the five essential lessons I have learned from running that are transferrable to implementing initiatives, conducting practice-based research and leading an antimicrobial stewardship program. To ensure this commentary will resonate with the reader, I provided an illustrative example connecting each lesson from running with a transferrable concept or practice in antimicrobial stewardship.

## Introduction

I took up running in mid-life, having always preferred tennis and squash. But when my father passed away from cancer shortly after his diagnosis, I began to run routinely to clear my head, perhaps subconsciously to manage my grief. Initially, running 2–3 km (under 2 miles) felt like a marathon. Over time, I built up enough stamina to participate in races, starting with 10 km (6.2 miles) and graduated to half-marathons (21.1 km or 13.1 miles). During a recent race, the idea of documenting the lessons I have learned from running that are transferrable to my work in antimicrobial stewardship (AMS) began to formulate. Below are the top five lessons that I hope will resonate with fellow runners and clinicians. See Table 1 for an illustrative example connecting each lesson with implementing or leading an AMS initiative. Let’s lace up our running shoes and get started!

**Table 1.** Applying the essential lessons I learned from running to leading in antimicrobial stewardship using an illustrative example of implementing an institution-wide antibiotic intravenous-to-oral (IV-to-PO) conversion protocol

Lesson from Running	What the lesson entails	Transferrable lesson applied to AMS	Example of implementing an IV-to-PO conversion protocol
1. Go slow…to go fast and far	• Over-training with running too far too fast puts us at risk of injuries • Avoid it by gradually increasing the distance and speed with frequent reassessment • Keep the end goal in mind	• Utilize the Model for Improvement and the PDSA cycle • Start with a small but well-defined scope • Collect and analyze baseline data using QI tools to identify the quality gap, the change idea and the intervention.^4^ • Iimplement it in a small scope before scaling up.	• Start with reviewing antibiotic prescribing data over a pre-defined period, identify the clinical areas (e.g. general medicine), common syndromes (e.g. community-acquired pneumonia or urinary tract infection) and antibiotic regimens suitable for the initial implementation of the IV-to-PO conversion protocol.
2. Setting goals and tracking performance metrics	• Runners track and analyze metrics: speed and splits, total mileage, cadence, elevation, nutrition, the shoes they wore • Some practice targeted exercise to address specific weaknesses, or train toward a specific time goal	• During the development of an AMS initiative, establish the aim statement, identify appropriate evaluative outcome, process and balancing measures • During initial implementation, collect user feedback and performance metrics to assess the feasibility of the initiative • Keep goal of initiative in mind to evaluate against pre-specified target	• During the pilot, assess feasibility of the protocol, e.g. automatic substitution for highly bioavailability antibiotics using electronic medical record system; pre-determined regimens by syndromes • Monitor for unintended consequences, e.g. patient who were unable to tolerate the PO switch • Scale up to other units after the pilot has been adequately assessed and potential issues addressed
3. I may be running alone but I am not alone in this race	• Running is both a solitary exercise and a communal experience • Helpers: coaches, pacers and supporters help runners stay motivated and safe	• Seek support from those who can foster optimal conditions or us to succeed, including organizational support, constructive feedback, advice and mentorship	• Identify an executive sponsor for resource allocation, organizational visibility and to ensure the goal of IV-to-PO conversion protocol aligns with the institution’s strategic goals • Invite key stakeholders to form the working group, e.g., internist, hospitalist, nursing representative, clinical pharmacist, dietitian, speech language pathologist. • Leverage the knowledge from the group to facilitate the success
4. Shake it off, I shake it off	• Picking ourselves up, dusting ourselves off after a setbackhelps to build to mental resilience • Although not all injuries can be easily shaken off, we should try to regain self-confidence to keep trying once we have recovered	• Leaders should foster a culture of resilience, learning from negative experiences with a growth mindset	• With the launch of a new initiative there may be pushback • Learning from challenging conversations in the moment can foster new skills and resilience.
5. Pace yourself	• Runners cherish the feeling of crossing the finishing line • Even though we run at different speeds, we all get there in the end • Pacing ourselves is the best way to ensure we can complete the course • Doing our best is enough	• Clinicians and researchers in an emerging field like AMS face a lot of pressure to compete against one another and succeed • Maintaining a healthy, balanced lifestyle is necessary to sustain a long career and prevents burn out	• A successful project may spur on additional ideas and QI initiatives • While exciting, it is important to balance workplace demands with maintaining a healthy lifestyle for a long and fulfilling career

## Lesson 1: Go slow…to go fast and far

One of the most common mistakes made by enthusiastic but inexperienced runners is running too fast too soon and increasing their mileage too quickly, assuming it will get them ready for a race sooner. But this approach can be counterproductive. Without a plan to condition the body to adapt to the biomechanics of running, overuse injuries such as plantar fasciitis, iliotibial band inflammation (“IT band syndrome”), knee pain and shin splints not only hamper the runner’s progress but also take the joy out of running. Rather, it is more conducive to start at a slower pace and run a shorter distance at first, then gradually increase the speed and distance with the end goal in mind.

This lesson is transferrable to the practice of AMS, which is founded under the auspices of healthcare quality improvement (QI).^1^ As a patient safety initiative, AMS aims to minimize the harms from antimicrobials, promote appropriate use, and mitigate against the emergence of antimicrobial resistance.^1–3^ The goal is to maintain the effectiveness of antimicrobials through sustainable improvements in prescribing practices. Under the Model for Improvement, the plan-do-study-act (PDSA) cycle encourages us to first invest the time to gather baseline data, analyze and identify the quality gap, before formulating the change idea and implementing the intervention.^4^ The concept of starting with a small but well-defined scope, identifying appropriate evaluative measures *a priori*, and assessing them adequately before scaling up for widespread impact is applicable to AMS. It also aligns with best practices in project management.^5^ Starting with the end point in mind, breaking down a large project (e.g. a new AMS intervention) into manageable steps, each appropriately resourced and monitored by frequent check-ins, help to ensure we are meeting the project milestones. Once an intervention has been refined and finalized, we can apply change management steps to facilitate implementation and adoption.^6^ In contrast, launching an initiative at full scale without any planned evaluation cycles may overwhelm key stakeholders such as prescribers, clinicians, patients, caregivers, and the AMS team. Potential consequences include delays, interruptions, missed opportunities, or unintended negative impact on patients and caregivers.

## Lesson 2: Setting goals and tracking performance metrics for continuous improvement

Initially, running a short distance felt physically and mentally challenging. To encourage myself, I continuously set small goals with small celebrations: “I will take a walking break after I reach *that stop sign*” or “I will have a drink of water after running up *this hill*.” Although trivial, these mini goals were achievable and celebrating those small wins buoyed me. As my endurance increased, the distance between those little goals grew longer. It was the culmination of achieving numerous small goals that got me through my first 10 km race. Additionally, setting a stretch goal – one that is on the cusp of attainability but requires dedicated effort, strategic training, and appropriate nutrition – serves as a long-term motivation for runners. To facilitate continuous improvement, runners are known for meticulously tracking their metrics, hence the burgeoning market of wearable devices and applications to log and analyze our runs. My current stretch goal is to complete a full marathon in a couple of years, having gained more experience with shorter distances. I will be following a training program with varying distance and speed, complemented by strength training and appropriate nutritional support for endurance.

Relating those experiences to leading an AMS program, under the Model for Improvement, establishing a program goal for the team and setting aims are essential to QI initiatives.^4,7^ The aim statement may be iterated through each PDSA cycle, but the fundamental domains remain consistent: 1) what is to be improved; 2) by when, i.e. time-bound; 3) by how much, i.e., measurable; 4) valid, i.e. how will one know an improvement has been made; and 5) where one can expect to see the improvement.^7^ Therefore, frequently evaluating performance metrics, i.e. outcome, process, and balancing measures appropriate for the AMS interventions facilitates success.^8^ Distinct from an aim, the program’s stretch goal should align with the strategic vision of the healthcare institution, yet sufficiently challenging such that the team is encouraged to seek novel change ideas or co-design interventions with stakeholders.^7,9,10^ Akin to appropriate nutritional support, the initiative should be adequately resourced.

## Lesson 3: I may be running alone but I am not alone in this race

For many, running is a solitary exercise to unclutter the mind. As I focus on putting one foot ahead of the other, I often come up with solutions to solve problems that have been swirling in my head. Running is also a communal experience. The comraderies from running groups and race participation improve our well-being beyond cardiovascular fitness. At a recent race, I came to appreciate the value of seeking out helpers and supporters, and connected this concept to my role as a leader in AMS. Who are the helpers and supporters? Some runners engage coaches to design strategic, targeted training exercises to improve their performance. At races, pacers are runners who complete the course at a steady pace and finish within a pre-designated time.^11^ Pacers are highly visible as they run with a sign that displays their speeds (e.g. “goal time: 4:40; pace: 10:40”) to guide runners at the pre-specified pace. As a group, pacers accommodate participants of different levels. Pacers are my “North Star” in a long-distance race because they help me gauge if I am running too fast or too slow for my capability. First responders are another group of helpers who are vital to the safety of all participants and spectators. Furthermore, I am always thankful for the volunteers handing out thousands of cups of water and energy bars to runners at the aid stations. Finally, spectators who cheer on the side are the biggest morale boosters, crucial in the last few miles of a long race when most runners are fatigued and will appreciate the motivation.

Leading in AMS, be it implementing a specific intervention or heading a full-fledged program, can at times feel like a pressurized, yet lonely endeavor. Seeking helpers and supporters (while avoiding detractors) can have a huge impact on the success of the AMS intervention and the program. It should be emphasized that this does not mean we only surround ourselves with individuals who are uncritical of our decisions or work. Rather, they are individuals who foster optimal conditions to help us succeed, which range from offering organizational support, constructive feedback, advice and mentorship.^12^ Additionally, as the leader of an AMS program, I consider it my responsibility to be a helper and supporter for team members, providing guidance, accountability and advocacy on their behalf for resources at the organizational level.^13^

## Lesson 4: “Shake it off, I shake it off!”

During a run last summer, I tripped and fell at the curb as I dashed across an intersection in the remaining seconds of the traffic light. Despite the pain and embarrassment, I quickly got up and moved off the road for safety. As I rinsed off my wounds, Taylor Swift’s popular “Shake It Off” sprang to mind: I must not let it affect my confidence in the upcoming race! Indeed, not all injuries can be shaken off easily, but my mishap prompted me to reflect on how I handled failures and rejections as a clinician, researcher and leader. There is much pressure in today’s culture to project and amplify personal successes, particularly on social media platforms. Surely, one should always celebrate one’s (and each other’s) achievements. But this quote from the American education scholar John Dewey “Failure is instructive. The person who really thinks learns quite as much from his failures as from his successes” encourages us to consider all experiences an opportunity for growth.

Putting this concept into practice, I often recount my early attempts to publish on the impact of AMS audit-and-feedback interventions in patients with acute leukemia to research learners. My manuscript was repeatedly rejected by academic journals, which was devastating as it is a topic I am passionate about, and I poured so much effort into the project. In hindsight, I realized that with each subsequent revision, I gained new knowledge and improved the manuscript for its eventual publication. Resilience, defined as the maintenance or quick recovery of mental health during or after a stressor exposure, emerged as a potential self-protective mechanism for healthcare workers against burnout during the pandemic.^14,15^ Healthcare leaders are well-positioned to inculcate resilience among themselves, team members, and promote organizational practices that support resilience within the healthcare system.^12,15,16^ Overcoming setbacks, learning from the experience and sharing it with others will help to advance us collectively.

## Lesson 5: Pace yourself

All runners at a race share a common goal: to experience the exhilaration of crossing the finish line! Individually, we may be running at different speeds, but we will all get there in the end. As each of us strives to achieve our “PB,” personal best time, it is essential to pace ourselves so that we do not run out of steam before the race is finished or get injured on the course. Furthermore, while runners are competing against one another, the emphasis of “personal best” reminds me that if I have done my utmost, it is sufficient. Relating this concept to leading in an emerging area such as AMS, as clinicians and researchers, we face constant pressure to compete and succeed. Maintaining an openness to learn, keeping a healthy, balanced lifestyle with strong social connections, and developing a positive attitude with self-efficacy will go a long way to sustain a long and impactful career.^16^

As a clinician, researcher and leader in AMS, there are many demands for my time. To strive for a work-life balance, I prioritize running by scheduling it into my weekly routine. Even running a short distance gives me a fresh perspective and approach to the challenges at work. Conversely, I also use my run to gain new knowledge by listening to podcasts on AMS/infectious diseases (e.g., the *ASHE* podcast), epidemiology, study design, or topics purely out of interest (e.g., history). I am fortunate that my family understands how important physical activity is to our well-being. It especially hit home during the COVID-19 pandemic, which happened to coincide with my graduate training in public health. Juggling multiple domains that straddle work and life certainly sharpened my focus to guard against mental burnout.

In summary, I have described the five lessons I learned from running and connected them to leading and implementing AMS. I hope this will encourage fellow runners and non-runners alike to share their reflection and insight into what their favorite meditative exercise or hobby has taught them. To the reader, if this commentary prompted you to take up running, a new exercise or hobby, even better. Have fun, be there to support the community and enjoy the camaraderie as you would with fellow stewards. Life is a journey: don’t forget to wave “hello” to each other along the way!
